# The Role of tRNA Fragments on Neurogenesis Alteration by H₂O₂-induced Oxidative Stress

**DOI:** 10.1007/s12031-025-02330-x

**Published:** 2025-04-11

**Authors:** Bilge Karacicek, Esra Katkat, Leman Binokay, Gunes Ozhan, Gökhan Karakülah, Sermin Genc

**Affiliations:** 1 Izmir Biomedicine and Genome Center, Izmir, Turkey; 2 Izmir Biomedicine and Genome Institute, Dokuz Eylul University, Izmir, Turkey; 3 Department of Neuroscience, Institute of Health Sciences, Dokuz Eylul University, Izmir, Turkey; 4 Department of Molecular Biology and Genetics, Izmir Institute of Technology, Izmir, Urla, Turkey

**Keywords:** TRFs, H_2_O_2_, NSCs, TRF-Glu-CTC, HyPer Zebrafish

## Abstract

Transfer RNAs (tRNAs) are small non-coding RNA molecules transcribed from tRNA genes. tRNAs cleaved into a diverse population tRNA fragments (tRFs) ranging in length from 18 to 40 nucleotides, they interact with RNA binding proteins and influence the stability and translation. Stress is one of the reasons for tRFs cleavage. In our study, we modeled oxidative stress conditions with hydrogen peroxide (H_2_O_2_) exposure and dealt with one of the frequently expressed tRF in the hippocampus region of the brain, which is tRF-Glu-CTC. For this purpose, neural stem cells (NSCs) were exposed to H_2_O_2_, and tRF-Glu-CTC levels were increased in various H_2_O_2_ concentrations. A decrease was seen in microtubule-associated protein 2 (MAP2) marker expression. To understand the H_2_O_2_ oxidative stress condition on the expression of tRNA fragments, 72 hpf zebrafish embryos exposed to different H_2_O_2_ concentrations, an increase in the level of tRF-Glu-CTC was observed in all concentrations of H_2_O_2_ compared to control. Subsequently, neurogenesis markers were figured out via Calb2a (calbindin 2a) in situ hybridization (ISH) and HuC/D immunofluorescence staining (IF) staining experiments. Under H_2_O_2_ exposure, a decline was observed in Calb2a and HuC/D markers. To understand the inhibitory role of tRF-Glu-CTC on neurogenesis, NSCs were transfected via tRF-Glu-CTC inhibitor, and neurogenesis markers (ßIII-tubulin, MAP2, and GFAP) were determined with qRT-PCR and IF staining. tRF-Glu-CTC inhibitor reversed the diminished neuronal markers expression under the exposure of H_2_O_2_. Gene Ontology (GO) enrichment analysis showed us that targets of tRF-Glu-CTC are generally related to neuronal function and synaptic processes.

## Introduction

tRNAs are the second most prevalent form of RNA in cells, a subclass of non-coding RNA (Mishima et al. 2014). tRNAs act as precursors in downstream cleavage events to produce functional tRFs. tRNAs undergo precision processing at the 5′- or 3′ end via endonucleases, like angiogenin (ANG) and Dicer, cleave pre- or mature tRNAs in certain situations to produce tRFs which is involved in post-transcriptional gene regulation (Tian et al. 2022). tRFs play crucial roles in various physiological functions of central nervous system (CNS) (Tian et al. 2022). There is a growing body of research indicating that both normal and diseased central nervous systems produce tRFs (Jehn et al. 2020; Fagan et al. 2021). Neurons and other cell types of CNS actively release tRFs, suggesting their significant role in the brain (Fagan et al. 2021). Jehn et al. (2020) conducted a study that examined the expression of tRFs distribution in brain. Investigation showed that 5′-tRFs were the most prevalent kind of tRFs in the cerebellum (82%), pre-frontal cortex (70%), and hippocampal regions (82%). The two most common 5′-tRFs in these brain regions were 5′-tRF-Glu-CTC and 5′-tRF-Gly-GCC (Jehn et al. 2020; Fagan et al. 2021). The role of tRNA processing abnormalities in neurodegenerative diseases has emerged as a critical area of research, revealing complex mechanisms that may promote conditions like Amyotrophic Lateral Sclerosis (ALS) and Parkinson’s disease (PD). Mutations in tRNA cleavage enzymes (ANG) have been reported in Amyotrophic Lateral Sclerosis (ALS) and PD (Greenway et al. 2006; van Es et al. 2011). Stressors alter the tertiary structures of mature tRNAs, which results in the cleavage of particular tRFs (Qin et al. 2020). Abnormal tRF expressions in neurological disease have been frequently observed, showing their potential as a biomarker (Qin et al. 2020). For instance, it has been reported that high levels of 5′-tRF-Val-CAC in ALS patients could be a prognostic biomarker for the slow course of the disease (Hogg et al. 2020).

Oxidative stress is a key factor leading to tRF formation and is also an environmental stressor affecting neurogenesis (Vergara et al. 2012). It is well recognized that H_2_O_2_ participates in signal transmission, which may affect the characteristics of stem cells. Intracellular H_2_O_2_ signaling is necessary for promoting self-renewal and neurogenesis in neural progenitor cells (NPCs) under physiological conditions. Elevated intracellular H_2_O_2_ levels induce an “activated” state in neural progenitor cells (NPCs), while lower levels restrict their proliferation. Moreover, it has been shown that neural stem cell populations in the brain require endogenous H_2_O_2_ synthesis (Perez Estrada et al. 2014). H_2_O_2_ is frequently utilized to study oxidative stress models in NPCs. In a study, H_2_O_2_ was applied at precisely controlled concentrations (50 μM and 100 μM) to investigate its effects on the differentiation of adult NPCs. The results revealed that NPCs exposed to H_2_O_2_ developed significantly higher numbers of neurons and oligodendrocytes compared to the unexposed control group (Perez Estrada et al. 2014).

Recent investigations have highlighted the impact of oxidative stress on tRNA cleavage and tRF production, revealing its crucial role in cellular stress responses (Rice 2011). A previous study demonstrated that oxidative stress induced by H_2_O_2_ triggered the initiation of tRNA cleavage and tRF production in PC-12 cells. In addition to H_2_O_2_, oxygen-glucose depletion was reported to significantly increase tRF production (Elkordy et al. 2018; Qin et al. 2020).

In our study, we aimed to investigate the role of tRF-Glu-CTC, which is one of the frequently expressed tRFs in human and primate brains (Jehn et al. 2020), in the process of neurogenesis in NSCs in vitro and zebrafish embryos in vivo.

We found that H_2_O_2_ induction increased the level of tRF-Glu-CTC in NSC and zebrafish embryos. Additionally, a reduction in the expression of neurogenesis markers was observed. To understand the role of tRF-Glu-CTC on neurogenesis, we performed a functional experiment using an inhibitor of tRF-Glu-CTC. We found that the tRF-Glu-CTC inhibitor reversed the diminished expression of neuronal markers under the exposure of H_2_O_2_, which supports its relationship with neurogenesis.

## Materials and Methods

### CGR8 Embryonic Stem Cell Culture

The murine embryonic stem cell line CGR8 was gifted to us by Prof. Marcel Leist (University of Konstanz, Germany). They were maintained in Dulbecco’s Modified Eagle Medium (DMEM) supplemented with 10% FBS, 2 mM Glutamax, 2 mM sodium pyruvate, 100 mM MEM nonessential amino acids (NEM-NEAA), and 50 µM β-mercaptoethanol. The medium was changed daily with freshly with 1000 U/mL murine leukemia inhibitory factor (mLIF) and 3i (2 µM SU5402, 800 nM PD184352, and 3 µM CHIR99021). CGR8 cells were passaged once in 2 days, cultured on 0.2% gelatin-coated T25 sterile flasks, and incubated at 37 °C in a humidified 5% CO_2_ incubator (Ying et al. 2003).

### Differentiation of CGR8 Cells to NSCs

For the differentiation of CGR8 (mouse embryonic stem cells-mESCs) into NSCs briefly, CGR8 cells were harvested with 0.05% trypsin. When they reached 80% confluency, 5 × 10^6^ cells in CGR8 medium with LIF were replated on 0.2% gelatin-coated T25 flask and incubated for 24 h in a humidified 5% CO_2_ incubator at 37 °C. The following day, 1.2 × 10^6^ cells/cm^2^ were plated on gelatin-coated dishes in N2B27 medium containing equal amounts of DMEM-F12 and Neurobasal‐medium, with N2 and B27 supplements, Glutamax, 100 µM β‐mercaptoethanol, 7.5 µg/mL insulin, 50 µg/mL bovine serum albumin (BSA), PenStrep; and incubated 7 days to obtain NSCs. During 7 days of differentiation, cell culture media were replaced on the second, fourth, and sixth days. At the end of differentiation, NSCs were detached with trypsinization and filtered through a 70 µm cell strainer to obtain single cells (Ying et al. 2003; Kleiderman et al. 2016).

### H_2_O_2_ Exposure of NSCs

H_2_O_2_ concentrations and the time period selection were determined by previously published studies in PC12 cells (Elkordy et al. 2018). Briefly, 1.2 × 10^6^ cells/cm^2^ were plated on gelatin-coated dishes, and after 7 days of differentiation, NSCs were exposed to H_2_O_2_ at a concentration of 50 μM for 6 h.

### Determination of the Expression of Neuronal Markers via qRT-PCR

Total RNA was extracted from NSCs using TRIzol reagents (Thermo Scientific, USA). The RNA concentration was assessed using a NanoDrop 2000 spectrophotometer (Thermo Scientific, Waltham, MA, USA). A high-capacity cDNA reverse transcription kit (Thermo Scientific, USA) was used according to the manufacturer’s instructions for cDNA synthesis from RNA. Quantitative real-time PCR (qRT-PCR) was performed using Promega GoTaq SYBR-Green master mix (Promega, USA) and ABI 7500 Real-Time PCR System (Thermo Scientific, USA) according to the manufacturer’s protocol. The primers used (ßIII-tubulin, MAP2, and GFAP) in experiments are given in Table 1. GAPDH was used as an endogenous control for qRT-PCR. Fold changes in expression were calculated using the 2^−ΔΔCt^ method (Zhang et al. 2019).

**Table 1:** The list of primers used for RT-qPCR

Gene	Primer sequence
ßIII-Tubulin	F TAGACCCCAGCGGCAACTAT R GTTCCAGGTTCCAAGTCCACC
MAP2	F GCCAGCCTCGGAACAAACA R GCTCAGCGAATGAGGAAGGA
GFAP	F GCCCGGCTCGAGGTCGAG R GTCTATACGCAGCCAGGTTGTTCTCT
GAPDH	F ACCACAGTCCATGCCATCAC R TCCACCCTGTTGCTGTA
tRNAGluCTC	F TCCCTGGTGGTCTAGTGGTTAGGATTCG R TTCCCTGACCGGGAATCGAACCCG
Anti-tRNAGluCTC	AGCGCCGAAUCCUAACCACUAGACCACCAGGGA
U6	F TGCTCGCTACGGTGGCACA R AAAACAGCAATATGGAGCGC

### Immunofluorescence Staining of Neuronal Markers in NSCs

**Fig. 1:**
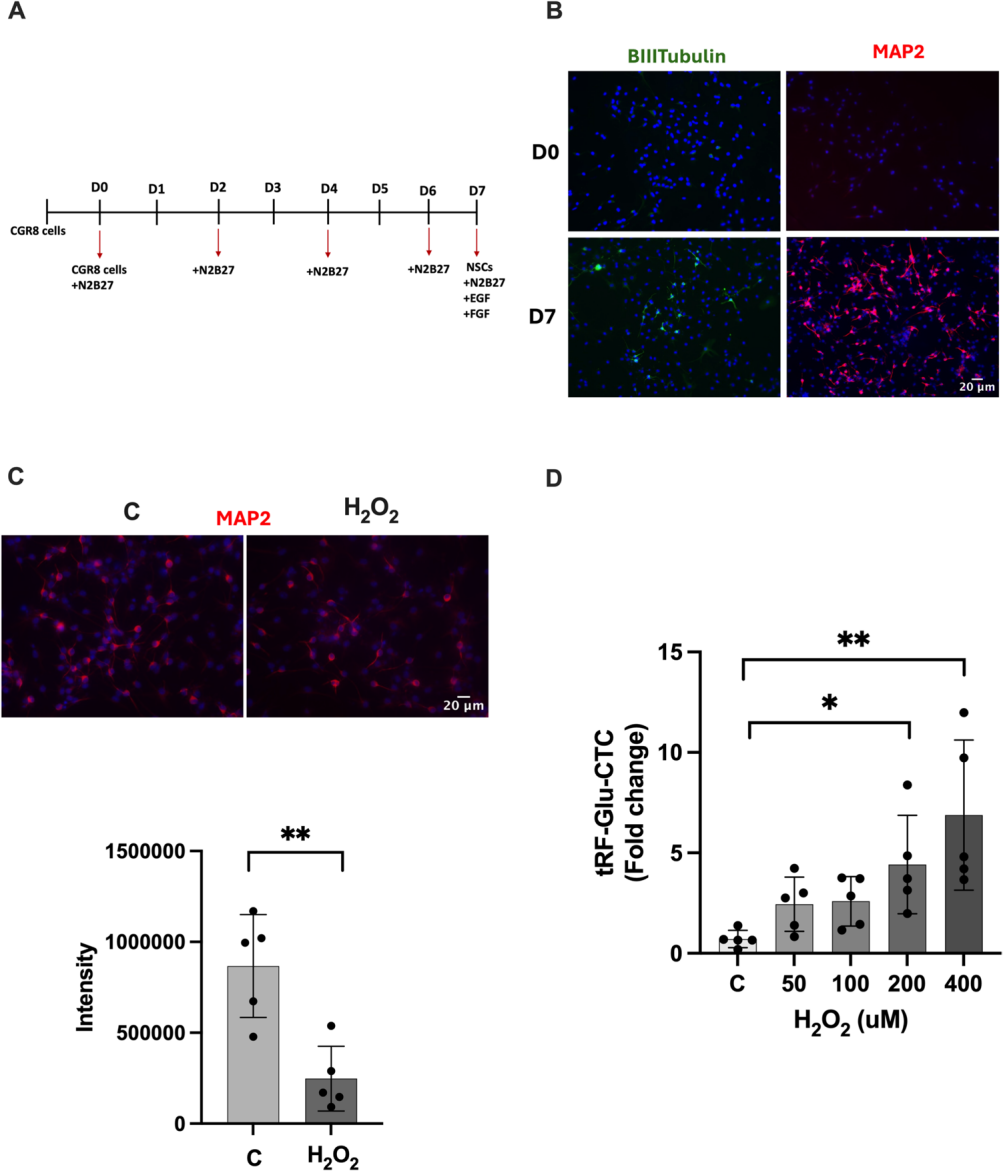
CGR8 neural differention to NSC and the changes of tRF-Glu-CTC level under different H_2_O_2_ concentrations.** A** Differentiation scheme of CGR8 into NSCs. **B** Immunofluorescence staining of neuronal markers(ßIII-tubulin and MAP2) at 7th day*,* images were taken 20 × magnification, Olympus IX61 fluorescent microscope). **C** Immunofluorescence staining of NSCs for neuronal marker (MAP2) after H_2_O_2_ exposure (images were taken 40 × magnification, Olympus IX61 fluorescent microscope) and intensity analysis (data are presented as mean ± SD, *n* = 5, Shapiro–Wilk test, **p < 0.01).** D** Changes in the levels of tRF-Glu-CTC in NSCs at different H2O2 concentrations (data are presented as mean ± SD, *n* = 5, one-way ANOVA Dunnett test, *p < 0.05, **p < 0.01)

For IF staining, NSCs were fixed with 4% paraformaldehyde for 30 min, followed by washing with PBS for 5 min twice. Next, permeabilization was performed with 0.2% Triton-X-100 for 45 min at room temperature (RT). After washing twice, blocking was achieved with 2% donkey serum blocking solution for 30 min at RT. Cells were stained overnight with primer ßIII-tubulin, MAP2, and GFAP antibodies at + 4 °C and later incubated with Alexa Fluor-488 and −594 conjugated secondary antibodies for 2 h at RT. Images were obtained via fluorescence microscopy at 20 × and 40 × magnification (Olympus IX61, Tokyo, Japan). The analysis of fluorescence intensity for MAP2 (Fig. 1C) was performed by selecting five regions of interest (ROIs) of equal size from both the C and H_2_O_2_ images. The intensity measurements of the ROIs were obtained using the ImageJ program.

### Transfection of NSCs with Inhibitor tRF-Glu-CTC Antisense 2′-OMe-RNA

On the 4th day of the 7-day protocol mentioned above, RNAiMAX and negative control (scramble- Silencer Select Negative Control No. 1 siRNA (Thermo Fisher) and tRF-Glu-CTC antisense 2′-OMe-RNA Glu-CTC inhibitor were applied (Jehn et al. 2020). After 50 μM H_2_O_2_ treatment for 6 h on day 3 of transfection, cells were collected, and RNA isolation and cDNA synthesis were performed. Afterward, qRT-PCR was performed using primers related to tRF-Glu-CTC and neurogenesis markers.

### Zebrafish Handling and Maintenance

Zebrafish were maintained according to the Izmir Biomedicine and Genome Center (IBG) guidelines under standard conditions, with a 12-h light–dark cycle at 28 °C, as described by the IBG Animal Care and Use Committee. The AB strain was used as the wild-type (wt) zebrafish (Audira et al. 2020) Tg(*actb2:hyper3*)*ka8* transgenic zebrafish line served as a reporter to quantify intracellular H_2_O_2_ levels in vivo (Bilan et al. 2013). Embryos were handled in E3 medium (5 mM NaCl, 0.17 mM KCl, 0.33 mM CaCl_2_, 0.33 mM MgCl_2_, 1% methylene blue) until 4 dpf at 28.5 °C incubator (Meyers 2018). Developmental staging of the embryos followed the criteria outlined (Kimmel et al. 1995). For H_2_O_2_ treatments (1 mM, 1.5 mM, and 2 mM), H_2_O_2_ was added into the E3 medium for 24 h in 72 h post-fertilization (hpf) zebrafish embryos.

### H_2_O_2_ Detection in Zebrafish Embryos

The HyPer zebrafish embryos were anesthetized in tricaine solution and embedded in low-melt agarose (1%) (Gauron et al. 2016). HyPer fluorescence was excited with 501/16 and 420/40 bandpass excitation filters, and the corresponding YFP emission was acquired using a 530/35 bandpass emission filter. Images were acquired at IBG Optic Imaging Facility using a Zeiss LSM880 confocal microscope in 20 × magnification (Carl Zeiss AG, Jena, Germany). HyPer ratios were calculated for each H_2_O_2_ concentration (1 mM, 1.5 mM, and 2 mM) via HyPer fluorescence signal intensity in 501/16 (as F_500_) divided to 420/40 (as F_420_) (Mishina et al. 2013). The size of the ROI was determined by including the brain region of the zebrafish in the image (the region above the eye, from this region equal three ROIs were taken, and average of ROIs used for measurements). The measurements were performed on five HyPer zebrafish embryos per group. For intensity measurements, Image J program is used.

### Determination of the Effects of H_2_O_2_ Induction on tRF-Glu-CTC Level in Zebrafish Embryos In Vivo by qRT-PCR

qRT-PCR was performed to determine the level of tRF-Glu-CTC in HyPer and wild-type AB zebrafish embryos. To determine the effect of H_2_O_2_ modulation on tRF-Glu-CTC level, 72 hpf transgenic zebrafish embryos were incubated for 24 h at 1 mM, 1.5 mM, and 2 mM H_2_O_2_ concentrations during the study. To examine the changes of tRF-Glu-CTC in the brain, the head region of the fish was separated from the body part by cutting the head region with the help of a scalpel after incubation. Forty head regions of zebrafish embryos were used for RNA isolation in RT-PCR experiments for each group. RNA isolation from the cut head regions was performed with QIAzol lysis reagent according to the manufacturer’s recommendations. The purity and concentration of isolated RNA samples were determined by spectrophotometric measurement in Nanodrop 2000. High-Capacity cDNA Reverse Transcription Kit (Thermo Scientific, USA) was used with 1 μg RNA for complementary DNA (cDNA) synthesis. After obtaining cDNA, quantitative PCR was performed on an ABI 7500 Fast quantitative PCR instrument using GoTaq® qPCR Master Mix (Promega; USA) according to the manufacturer’s instructions. The relevant tRNA fragment primer sequences are given in the table below (Table 1) (Chen et al. 2021). U6 was used as an internal control. Fold changes in expression were calculated using the 2^−ΔΔCt^ method.

### RNA Probe Synthesis and Whole-Mount In Situ Hybridization

Specific primers were designed using Primer-BLAST from NCBI to obtain antisense RNA probes targeting particular mRNAs, with the T7 RNA polymerase promoter sequence added to the 5′ end of each reverse primer (Ye et al. 2012). The PCR-based templates were generated from 5 dpf zebrafish larval cDNA and were purified using NucleoSpin Gel and PCR Clean-up Kit (Macherey–Nagel, Duren, Germany). Digoxigenin (DIG)-labeled antisense RNA probes were synthesized and labeled with DIG RNA labeling mix (Merck & Co., Inc., NJ, United States) by in vitro transcription T7 RNA polymerase kit (Thermo Fisher Scientific, MA, United States). The in vitro transcription RNA probes were purified using the RNA Clean and Concentrator-25 kit (Zymo Research, CA, United States). For whole-mount in situ hybridization (WMISH), zebrafish larvae were fixed at 4 dpf in 4% PFA (in 1 × PBS) overnight. WMISH was conducted using an antisense probe for *Calb2a* as described (Thisse and Thisse 2008). After overnight fixation, embryos were treated with 3% H₂O₂/0.5% KOH for up to 1 h to remove pigmentation. Following bleaching, embryos were washed with 1X PBS and dehydrated through serial incubations in 25%, 50%, and 75% (vol/vol) methanol in PBT (1 × PBS, 0.1% Tween-20). Embryos were then stored overnight at − 20 °C in 100% methanol. The next day, embryos were rehydrated through serial incubations in 75%, 50%, and 25% (vol/vol) methanol in PBT, followed by three 5-min washes in 100% PBT. Permeabilization was performed using proteinase K (10 µg/mL) at RT for 10 min, and digestion was stopped by incubating embryos in 4% PFA for 20 min. Residual PFA was washed off with 1 × PBT. Embryos were prehybridized in hybridization buffer (50% deionized formamide, 5X SSCT, 50 µg/mL heparin, 500 µg/mL RNase-free tRNA, 1 M citric acid, pH 6) at 67 °C for 3 h in a water bath. The buffer was then replaced with a hybridization mix containing RNA probe (1 ng/µL), and embryos were incubated overnight at 67 °C. The next day, the hybridization mix was removed, and embryos were washed sequentially in hybridization buffer, 50% 2 × SSCT in formamide, 2 × SSCT, 0.2 × SSCT, and 1 × PBT at 67 °C. Blocking was performed for 1 h at RT in blocking buffer (1 × PBT, 5% sheep serum (vol/vol), 10 mg/mL BSA). Embryos were then incubated overnight at 4 °C with anti-digoxigenin antibody (1:4000, 11333089001, Roche) in blocking buffer. The following day, embryos were washed in 1 × PBT and stained using NTMT buffer containing nitro blue tetrazolium (NBT, 0.175 µg/mL) and 5-bromo 4-chloro 3-indolyl phosphate (BCIP, 0.175 µg/mL). Images were captured using a SZX10 Olympus stereomicroscope. The intensity measurements for Calb2a were performed by averaging three ROIs for each zebrafish in the groups. The measurements were performed on five zebrafish embryos per group, and intensity is measured via Image J program.

### Whole-Mount Immunofluorescence Staining of Zebrafish Larvae

The immunofluorescence staining was performed as described previously (Martinez-Lopez et al. 2021). Larvae were fixed in 4% paraformaldehyde (PFA) in 1 × PBS overnight at 4 °C and washed with 1 × PDT (1 × PBST, 0.3% Triton-X, 1% DMSO). Fixed larvae were permeabilized with ice-cold acetone at − 20 °C for 7 min, and then larvae were blocked for 1 h in blocking buffer (10% bovine serum albumin, 1% DMSO, 0.3% Triton-X, 15 µL/1 mL goat serum). The primary antibody incubation was performed at 4 °C overnight. The next day, the larvae were washed several times with 0.1% Triton-X in PBS and subjected to secondary antibody incubation for 2 h at RT. Larvae then were washed with 0.1% Triton-X in PBS mounted in 80% glycerol between two coverslips and stored at 4 °C. The antibodies used mouse anti-HuC/D Antibody (#A-21271, Thermo Fisher Scientific, MA, United States) and Cy™5 AffiniPure™ Donkey Anti-Mouse IgG (715–175-150, Jackson Immunoresearch Laboratories, PA, United States). Nuclear staining was carried out using 4′,6-diamidino-2-phenylindole (DAPI; 4083S, Cell Signaling Technology, MA, United States). Larvae were imaged using a Zeiss LSM880 fluorescence confocal microscopy (Carl Zeiss AG, Jena, Germany). Confocal images were captured with a 25 × objective lens using the z-stack function with a 10 µm interval between each slice. For HuC/D intensity measurements, an ROI is used for each zebrafish. The ROI is indicated on the zebrafish image, marked with yellow square. The measurements were performed on five zebrafish embryos per group, and Image J program is used for the intensity analysis.

### Target Analysis and Gene Ontology (GO) Analysis

The identification of potential targets for tRF-Glu-CTC (tRFdb-5022a tRF ID for tRF-Glu-CTC in tRFtarget) was accomplished utilizing the tRFtarget platform (http://trftarget.net). Subsequently, to elucidate the functional implications of these targets, we conducted GO analysis. These analyses were performed using the clusterProfiler package (version 4.10.1) within the R environment. The results of these analyses were visually represented through a bar plot for GO analysis.

To further investigate the interrelationships between tRFdb-5022a and its targets, we constructed an interaction network. This network was generated using the igraph package (version 2.0.3) in R, where tRF and its target genes were depicted as nodes, with the interactions between them represented as edges. The network visualization was done by using the ggraph package (version 2.2.1).

### Statistical Analysis

Data analysis was conducted using IBM SPSS Statistics 29.0 software program and GraphPad version 10.2.3 (GraphPad Software Inc., CA, USA). The data were presented and analyzed according to distribution characteristics. In the compared groups, the number of “n” and the within-group variance was small, and the suitability for parametic distribution was analyzed with the Shapiro–Wilk test.

Parametric data were shown with the mean ± standard deviation (SD) graph, and non-parametric data were shown with the boxplot graph. In the comparison of two independent groups that were normally distributed (parametric condition), the t-test was used, and in the data that did not provide the assumption of normal distribution, the non-parametric Mann–Whitney U test was used. In the comparison of three independent groups, parametric one-way ANOVA F test, post hoc Dunnet test, non-parametric Kruskal–Wallis H test, and post hoc Dunn’s test were applied. A significance threshold of *p* < 0.05 was applied to all analyses.

## Results

### The Role of H_2_O_2_ on tRF-Glu-CTC Level in NSCs

We decided to use mouse neural stem cells to understand the role of H_2_O_2_ oxidative stress on tRF-Glu-CTC levels in NSCs. Firstly, mouse embryonic stem cells, CGR8 cells, were differentiated into mouse neural stem cells (Fig. 1A). IF staining was performed at days 0 and 7 for ßIII-tubulin (early neuronal differentiation marker) and MAP2 (mature neuronal marker) differentiation markers. In both markers, an increase was seen on day 7 according to day 0 (Fig. 1B). Under the H_2_O_2_ stress condition, the MAP2 marker diminished according to control (Fig. 1C, intensity analysis Shapiro–Wilk test, *p* = 0.003(**p < 0.01)). Through the differentiation, distinctive staining of neuronal markers was seen in NSCs at 7 days. Later, the level of tRF-Glu-CTC under various H_2_O_2_ concentrations in NSCs was evaluated. Varied concentrations of H_2_O_2_ (50–400 μM) were added to the media for 6 h. Although an increase was observed at all concentrations, a significant increase was found at 50 μM and 400 μM H_2_O_2_ concentrations in the level of tRF-Glu-CTC according to the control (Fig. 1D, one-way ANOVA Dunnett test, C vs. 200 μM *p* = 0.045(*p < 0.05), C vs. 400 μM *p* = 0.01(**p < 0.01)).

#### H_2_O_2_ Dynamics of HyPer Zebrafish Embryos

**Fig. 2:**
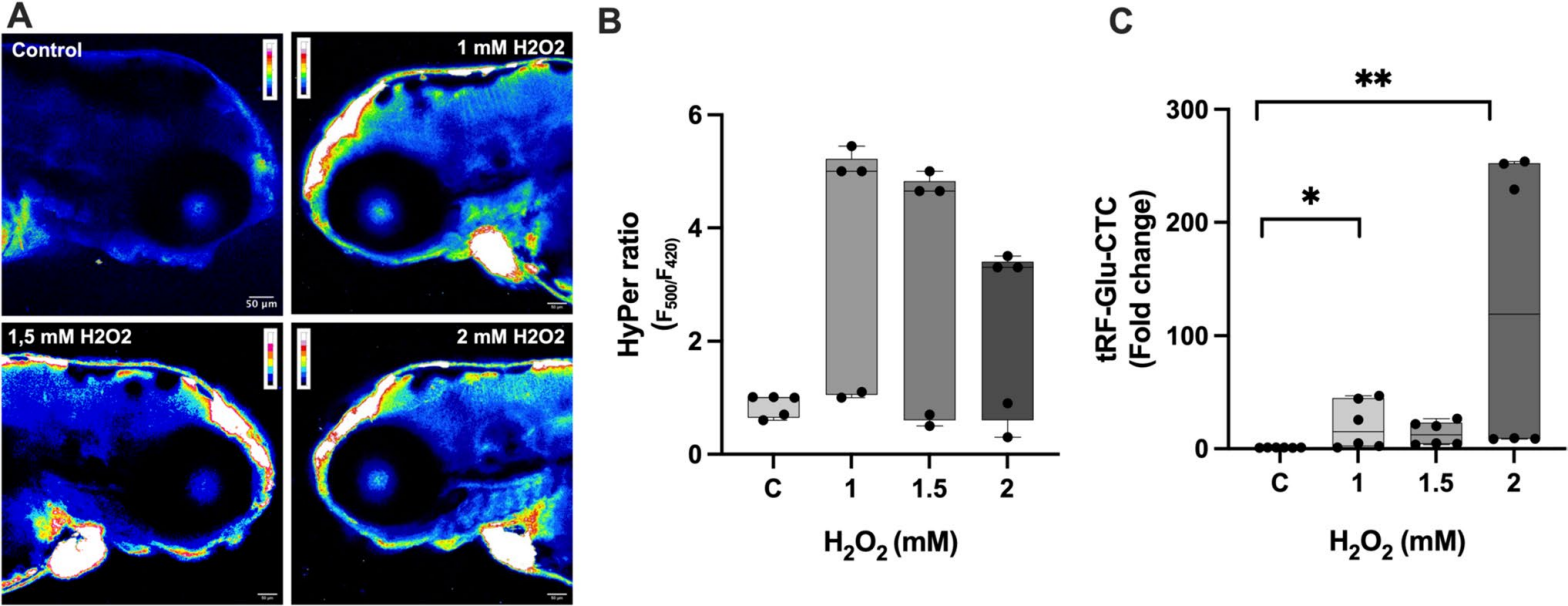
Effect of in vivo HyPer zebrafish H_2_O_2_ exposure on tRF-Glu-CTC levels.** A** H_2_O_2_ levels in 72 hpf HyPer zebrafish embryos for 24 h. HyPer imaging for different concentrations of H_2_O_2_. The H_2_O_2_ levels are inferred from the F_500_/F_420_ excitation ratio of HyPer. Respectively, control, 1 mM, 1.5 mM, and 2 mM H_2_O_2_. **B** Quantification of the H_2_O_2_ levels in the control and H_2_O_2_ concentrations in 72 hpf HyPer zebrafish embryos; images were taken 20 × magnification, Zeiss LSM 880 confocal microscopy (data are presented as boxplot, *n* = 5*, ns,* Kruskal–Wallis test, *p* = 0.168). **C** Changes in levels of tRF-Glu-CTC fragment in HyPer 72 hpf embryos after 24 h H_2_O_2_ exposure (data are presented as boxplot, *n* = 6, Kruskal–Wallis test, *p < 0.05, **p < 0.01)

To assess the intracellular levels in the presence of exogenous H_2_O_2_ concentrations, live imaging was performed in 72 hpf Tg(actb2:hyper3)ka8 zebrafish embryos with a confocal microscope. The brain area of the embryos was observed for 1 mM, 1.5 mM, and 2 mM H_2_O_2_ concentration. In our previous study, we showed that these concentrations affect neurogenesis (Engur et al. 2023). In 72 hpf HyPer embryos for 24 h H_2_O_2_ induction regarding control, the highest HyPer ratio was observed in 1 mM H_2_O_2_ concentration, and also an increase was observed in 1.5 mM and 2 mM H_2_O_2_ concentrations (Fig. 2A, B and Fig. 2B, Kruskal–Wallis test, *p* = 0.168).

#### tRF-Glu-CTC Fragment Level Changes in Various H_2_O_2_ Concentrations

Also, one of the most abundantly expressed tRNA-derived fragments in the brain, tRF-Glu-CTC (Jehn et al. 2020), was determined for 1 mM, 1.5 mM, and 2 mM H_2_O_2_ concentrations on 72 hpf HyPer zebrafish embryos via qRT-PCR. The tRF-Glu-CTC level was increased in all concentrations of H_2_O_2_ according to the control, but the highest tRF-Glu-CTC level was observed in 2 mM H_2_O_2_ concentration (Fig. 2C top, Kruskal–Wallis test, C vs. 1 mM *p* = 0.048 (*p < 0.05), C vs. 2 mM *p* = 0.001(**p < 0.01).

### The Role of H_2_O_2_ Oxidative Stress on tRF-Glu-CTC Level and Neurogenesis In Vivo AB Wild-Type Zebrafish

**Fig. 3:**
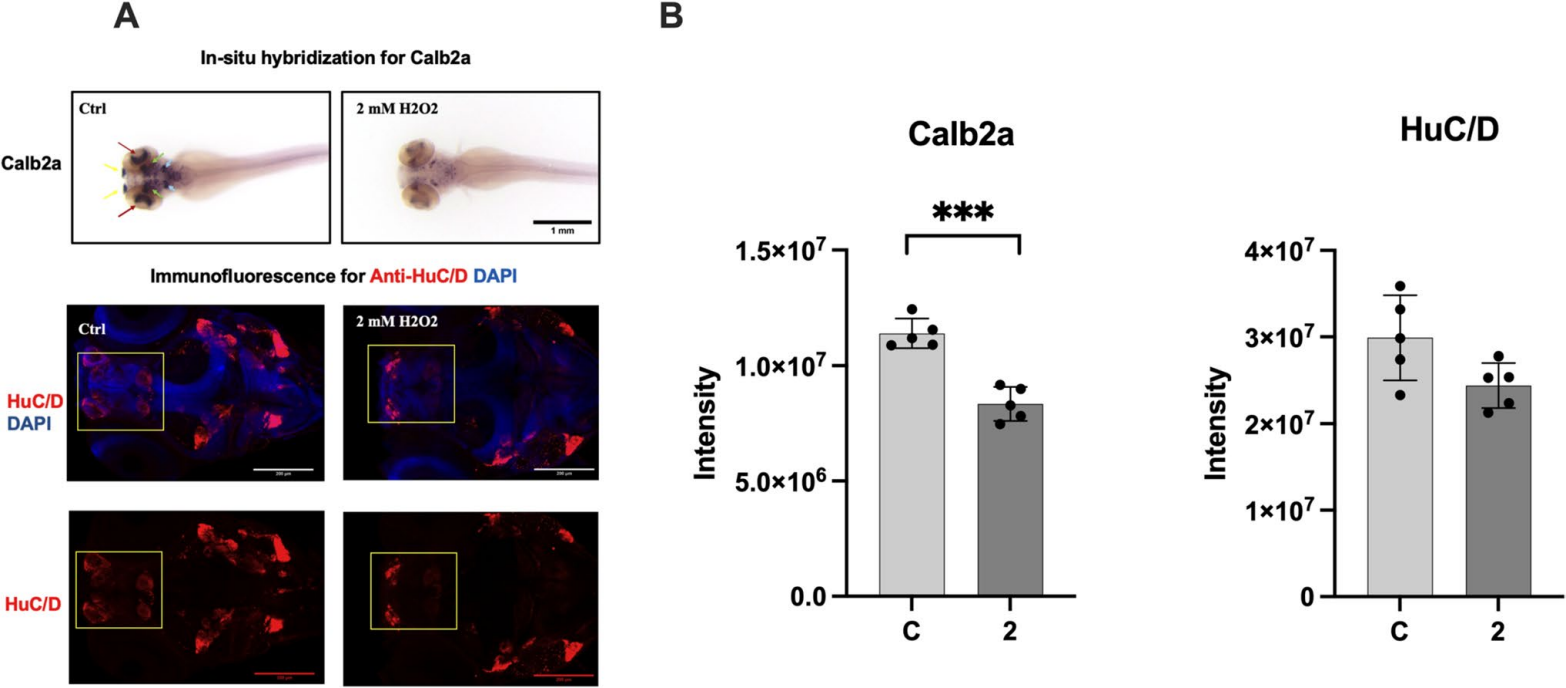
Effect of in vivo AB wild-type zebrafish H_2_O_2_ exposure on tRF-Glu-CTC neurogenesis. Role of H_2_O_2_ on neurogenesis. **A** In situ hybridization (Calb2a) and immunofluorescence (HuC/D) images of in wild-type AB zebrafish 72 hpf embryos after 24 h H_2_O_2_ exposure. Calb2a expression decreases in various cranial structures, including the olfactory bulb (yellow arrows), retina (red arrows), cerebellum (green arrows), and hindbrain (blue arrows). Anti-HuC/D (red) staining of the heads of zebrafish larvae obtained from confocal microscopy. Yellow labels indicate telencephalon and habenula, respectively. **B** Intensity analysis of Calb2a and HuC/D (Data are presented as mean ± SD, *n* = 5, t-test, for Calb2a ***p < 0.001 and HuC/D* p* = 0.057). C, control; 2, 2 mM H_2_O_2_

We used 72 hpf of the wild-type AB zebrafish embryos and were exposed to 24 hp H_2_O_2_ by adding their water. The H_2_O_2_ concentration of 2 mM was chosen for the wild-type AB zebrafish embryos because the highest tRF-Glu-CTC levels were seen in HyPer zebrafish embryos. To figure out the role of 2 mM H_2_O_2_ on neurogenesis in wild-type AB zebrafish embryos, ISH with Calb2a and IF staining with HuC/D were performed (Fig. 3A). A decreasing trend was observed in both neurogenesis markers (Calb2a and HuC/D) compared to the control. We measured the intensities for both of the markers. A statistically significant reduction in Calb2a level was observed (Fig. 3B, Calb2a t-test, *p* = 0.001(***p < 0.001) and HuC/D, t-test, *p* = 0.057).

The ISH analysis performed on 4 dpf zebrafish larvae revealed a noticeable reduction in the expression of Calb2a, a protein abundantly expressed in neurons, in several cranial structures, including the olfactory bulb, retina, cerebellum, and hindbrain. Immunofluorescence staining for HuC/D, a neuronal marker, was conducted on 4 dpf zebrafish larvae to visualize the neuronal cells. Moreover, we observed a significant decrease in neuronal differentiation in the brain regions of crispants, as evidenced by immunofluorescence staining with the anti-HuC/D antibody, which marks neuronal cell bodies. The control group (Ctrl) displays a robust HuC/D expression, while the larvae treated with 2 mM H_2_O_2_ exhibit a marked decrease in HuC/D positive neuronal cells, specifically within the telencephalon region.

These results collectively demonstrate that oxidative stress, induced by H_2_O_2_ treatment, adversely affects both the expression of Calb2a and the presence of HuC/D positive neurons in 4 dpf zebrafish larvae.

### In Vitro Effects of tRF-Glu-CTC Inhibition on Neurogenesis in NSCs

**Fig. 4:**
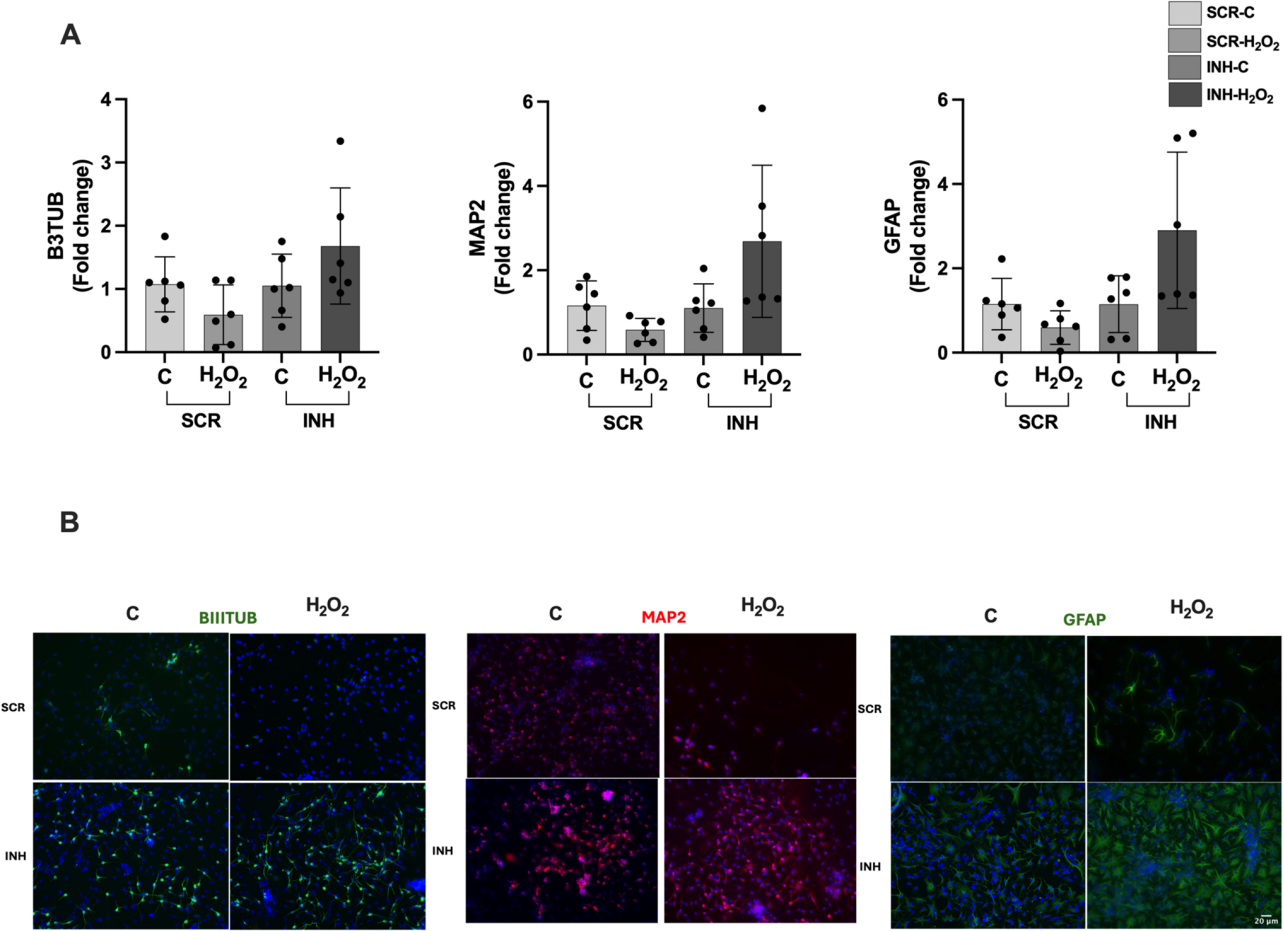
Role of tRF-Glu-CTC inhibition on neurogenesis of NSCs. **A** Expression levels of neurogenesis markers (ßIII-tubulin, MAP2, and GFAP) after transfection of SCR and tRF-Glu-CTC antisense 2′-OMe-RNA (inhibitor) in 50 μM H_2_O_2_ for 6 h for neurogenesis markers (data are presented as mean ± SD, *n* = 6, t-test, BIIITUB t-test, C-SCR vs. H_2_O_2_-SCR *p* = 0.097 and C-INH vs. H_2_O_2_-INH *p* = 0.171, MAP2 t-test, C-SCR vs. H_2_O_2_-SCR *p* = 0.067 and C-INH vs. H_2_O_2_-INH *p* = 0.054, GFAP t-test, C-SCR vs. H_2_O_2_-SCR *p* = 0.090, C-INH vs. H_2_O_2_-INH, Mann–Whitney test *p* = 0.180). **B** Immunofluorescence images of neurogenesis markers (ßIII-tubulin, MAP2, and GFAP) after transfection (images were taken 20 × magnification, Olympus IX61 fluorescent microscope). INH, inhibitor; SCR, scramble

To confirm the role of tRF-Glu-CTC under exposure of H_2_O_2_ on neurogenesis in NSCs, inhibitor of tRF-Glu-CTC was used, and the neurogenesis markers were assessed via qRT-PCR and IF staining. ßIII-tubulin (immature neuron marker), MAP2 (mature neuron marker), and GFAP (astrocyte marker) were used as neurogenesis markers. mRNA levels of neurogenesis markers were decreased in H_2_O_2_ condition according to control (Fig. 4A, BIIITUB t-test, C-SCR vs. H_2_O_2_-SCR *p* = 0.097 and C-INH vs. H_2_O_2_-INH *p* = 0.171, MAP2 t-test, C-SCR vs. H_2_O_2_-SCR *p* = 0.067 and C-INH vs. H_2_O_2_-INH *p* = 0.054, GFAP t-test, C-SCR vs. H_2_O_2_-SCR *p* = 0.090, C-INH vs. H_2_O_2_-INH, Mann–Whitney test *p* = 0.180). Also, the IF staining for neurogenesis markers supports the qRT-PCR results (Fig. 4B). Briefly, tRF-Glu-CTC inhibitor reversed the diminished neuronal markers expression under the exposure of H_2_O_2_ (Fig. 4A). This can be explained as tRF-Glu-CTC has inhibitory roles on neurogenesis under H_2_O_2_ exposure.

### GO Enrichment Analysis for tRF-Glu-CTC Target Genes

The potential target genes of tRF-Glu-CTC (tRFdb-5022a) were identified using the tRFtarget platform which predicts tRF-target interactions based on sequence complementarity and binding free energy. From this platform, we obtained a ranked list of genes with the strongest predicted interactions for further analysis.

**Fig. 5:**
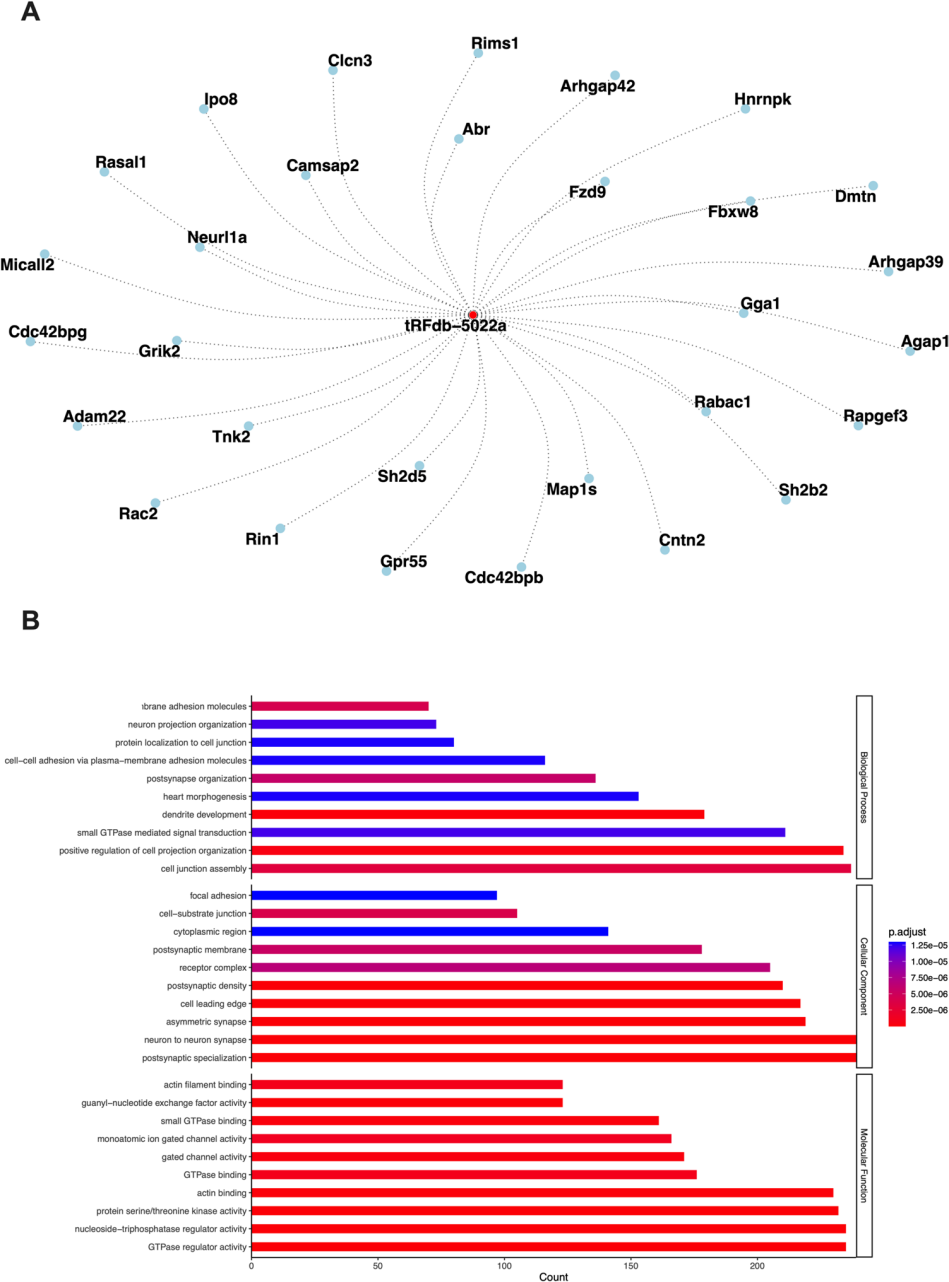
Interaction network and GO analysis of tRF-Glu-CTC target genes.** A** Network visualization of the top 30 predicted target genes of tRF-Glu-CTC (tRFdb-5022a), generated using the igraph and ggraph packages in R. tRF-Glu-CTC is shown at the center of the network, with its connections (edges) to target genes displayed as dotted lines.** B** GO enrichment analysis results for the predicted target genes of tRF-Glu-CTC under three classifications: biological process (BP), molecular function (MF), and cellular component (CC). Results indicate significant enrichment of terms associated with neuronal function and synaptic processes

To better understand the relationships between tRF-Glu-CTC and its predicted targets, we generated an interaction network consisting of the top 30 potential target genes (Fig. 5A, tRFdb-5022a is tRF ID for tRF-Glu-CTC). The interaction network was constructed using the igraph and ggraph packages in R, where tRF-Glu-CTC and its target genes were represented as nodes, and their predicted interactions were depicted as edges. At the center of the network, tRF-Glu-CTC is depicted as the red node, symbolizing its regulatory role as the core molecule influencing the surrounding target genes. The blue nodes represent its 30 predicted target genes, with each node corresponding to a specific gene.

Among 30 potential genes Arhgap39 involved in postsynaptic organization (Nowak 2018), CNTN2 in axon guidance (Savvaki et al. 2021), Adam22 in neurogenesis and myelination (Ozkaynak et al. 2010), Map1s in neuron projection (Orban-Nemeth et al. 2005), Neurl1a in neuronal synaptic plasticity (Whalley 2012), and Grik2 in neurophysiological process (Guzman et al. 2017).

The dotted lines connecting tRF-Glu-CTC to its target genes indicate the predicted regulatory interactions, which may represent pathways through which tRF-Glu-CTC exerts its influence. This network underscores the biological significance of tRF-Glu-CTC in regulating genes associated with critical neuronal functions such as synaptic plasticity, axon guidance, and neurogenesis, suggesting its potential role in neuronal development and connectivity.

To further elucidate the biological significance of the predicted target genes of tRF-Glu-CTC, we performed GO enrichment analysis based on three different categories: biological process (BP), molecular function (MF), and cellular component (CC) (Fig. 5B). Ten GO terms for biological processes with cell junction assembly being the highest percentage, ten GO terms for molecular functions GTPase regulator activity, nucleoside-triphosphatase regulator activity, and protein serine/threonine kinase activity being the highest percentage. Lastly, ten GO terms for cellular components with postsynaptic specialization, neuron to neuron synapse, and asymmetric synapse being the highest percentage were found. GO terms for tRF-Glu-CTC showed us that, generally, its targets are related to neuronal function and synaptic processes.

The combined results of the network analysis and GO enrichment analysis provide strong evidence that the target genes of tRF-Glu-CTC are closely associated with neuronal development, synaptic organization, and neurotransmission processes. The network analysis highlights key genes involved in postsynaptic organization, axon guidance, neurogenesis, and synaptic plasticity. These findings suggest that tRF-Glu-CTC may act as a regulatory molecule influencing critical pathways in neuronal function and synaptic connectivity.

## Discussion

Our results showed that H_2_O_2_-induced stress condition resulted in increased expression of tRF-Glu-CTC level in in vivo (zebrafish) and in vitro (NSC). Additionally, neurogenesis is negatively affected both in zebrafish embryos and NSCs, and a decrease is seen in neurogenesis markers. The inhibitor of tRF-Glu-CTC reversed the diminished neurogenesis markers expression under the exposure of H_2_O_2_.

In our study, we modeled oxidative stress by using H_2_O_2_. We used 72 hpf transgenic HyPer zebrafish embryos to examine the H_2_O_2_ dynamics in various H_2_O_2_ concentrations in the brain region of embryos. HyPer ratio increased at all concentrations compared to control, however started to decrease after 1 mM H_2_O_2_ concentration. This may be due to the saturation of HyPer probes. Also, transgenic zebrafish sensitive to H_2_O_2_ detection with the HyPer probe has been used to figure out highly variable spatiotemporal patterns of H_2_O_2_ concentrations during zebrafish development (Gauron et al. 2016).

Intracellular H_2_O_2_ signaling is critical for mediating self-renewal and neurogenesis in NPCs under physiological conditions (Perez Estrada et al. 2014). We found that in NSC’s seven-day differentiation protocol, at the end of the 7 days, an increase was observed in neurogenesis markers (MAP2 and ßIII-tubulin). When NSCs were exposed to H_2_O_2_, a decline was observed in neurogenesis markers. The effect of H_2_O_2_ on differentiation appears to be dose-dependent.

An increase in the differentiation marker (ßIII-tubulin) was observed in long-term and high-dose treatments (Perez Estrada et al. 2014), whereas a reduction was reported in low-dose and short-term applications, similar to our study (Perez Estrada et al. 2014; Eltutan et al. 2022). Following the in vitro experiments conducted with NSCs, in vivo evaluations were performed on wild-type AB zebrafish embryos to assess neurogenesis markers. In wild-type AB zebrafish embryos under H_2_O_2_ exposure, a reduction in Calb2a and HuC/D neurogenesis markers was observed, similar to that of NSCs. These changes in the distribution of neuronal cell bodies around the outer layers of the zebrafish larvae brain could indicate disrupted neurogenesis which is revealed by a significant reduction in staining intensity of HuC/D in the telencephalon region and the cerebellum at the hindbrain-midbrain boundary of the crispants. tRF-Glu-CTC is a frequently expressed tRFs in the hippocampus, indirectly affecting brain development and neurogenesis (Jehn et al. 2020; Zhang et al. 2023).

Besides studies on stress conditions with tRF-Glu-CTC in the aging brain (Li et al. 2024) and neurodegenerative disorders (Wu et al. 2021), research has also investigated its implications in cancer. Under stress conditions in breast cancer (Cui et al. 2019), significant upregulation of tRF-Glu-CTC was observed (Cui et al. 2019).

In our study, we found an increase in tRF-Glu-CTC expression and neurogenesis inhibition in H_2_O_2_-induced stress conditions. To understand the inhibitory role of tRF-Glu-CTC on neurogenesis, NSCs were transfected via tRF-Glu-CTC inhibitor, and neuronal markers changes (ßIII-tubulin-Tubulin Beta 3 Class, MAP2, and GFAP-glial fibrillary acidic protein) were determined with qRT-PCR and IF staining. tRF-Glu-CTC inhibitor reversed the diminished neuronal markers expression under the exposure of H_2_O_2_, supporting its inhibitory role on NSC differentiation.

It has been reported that the tRF-Glu-CTC fragment targets genes involved in neuronal processes in a small RNA sequencing study of primate hippocampal tissues. When the expression profiles in the sequencing were examined, it was shown that the transcripts targeted by tRF-Glu-CTC are involved in neuronal processes such as axon growth and neuronal differentiation (Jehn et al. 2020). In our study, we also analyze the GO enrichment analysis provided via the tRForest database (https://trforest.com/) to explore the biological functions of biological process (BP), molecular function (MF), and cellular component (CC). Our studies GO terms for the CC included “neuron to neuron synapse,” “postsynaptic membrane,” “postsynaptic density,” “asymmetric synapse,” and “postsynaptic specialization” and for the BP included “dendrite development,” “neuron projection organization,” and “postsynapse organization” are corresponding terms with neuronal processes. In addition, MF, BP, and CC GO terms related to “small GTPase” is essential in neurogenesis and brain development (Endo and Cerione 2022) which shows tRF-Glu-CTC is related neuron to neuronal processes.

Our study has several limitations that need further investigation. For instance, our research focused solely on zebrafish embryos at 72 hpf, leaving out other tRFs associated with neurogenesis and different developmental stages. Additionally, future studies should explore a range of H_2_O_2_ concentrations and exposure durations in vitro to better understand its effects. Identifying and analyzing other tRFs and targets to neurogenesis is also essential for advancing our knowledge.

In conclusion, our findings indicate that exposure to H_2_O_2_ increases tRF-Glu-CTC expression while suppressing neurogenesis in NSCs and zebrafish embryos. Moreover, inhibiting tRF-Glu-CTC reversed the expression of neurogenesis markers, suggesting a potential inhibitory role of tRF-Glu-CTC on NSCs. Further research is needed to elucidate the role of other tRFs in neurogenesis under stress conditions.

## Data Availability

No datasets were generated or analysed during the current study.
